# Supplementary figures and images for: Dimeric R25CPTH(1–34) activates the parathyroid hormone-1 receptor in vitro and stimulates bone formation in osteoporotic female mice
Source: eLife. 2025 Mar 28;13:RP97579. doi: 10.7554/eLife.97579 (PMC11952747; doi:10.7554/eLife.97579)

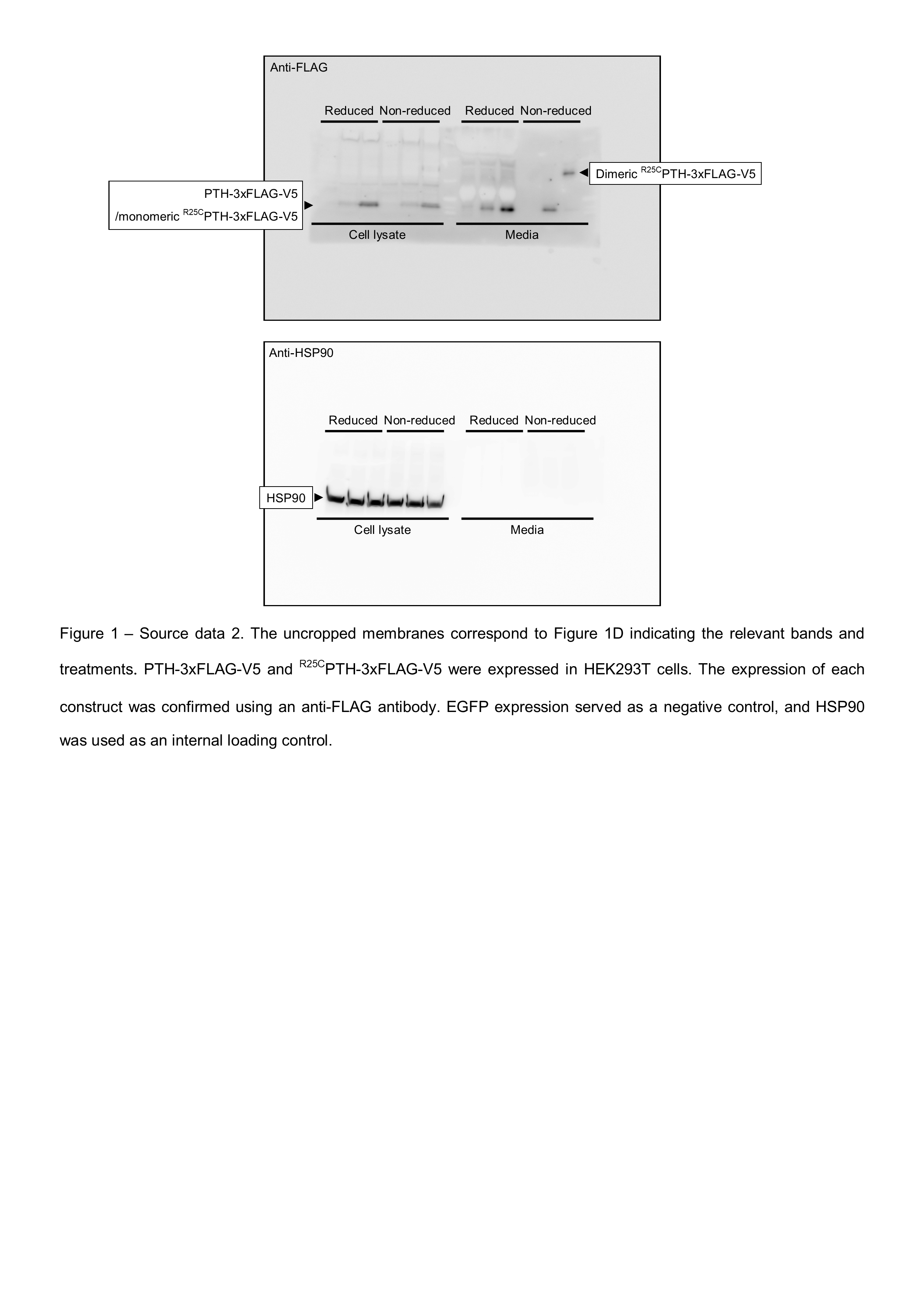

Supplement: Figure 1—source data 2. [file elife-97579-fig1-data2.zip › Figure 1D_Source data 2.tif]

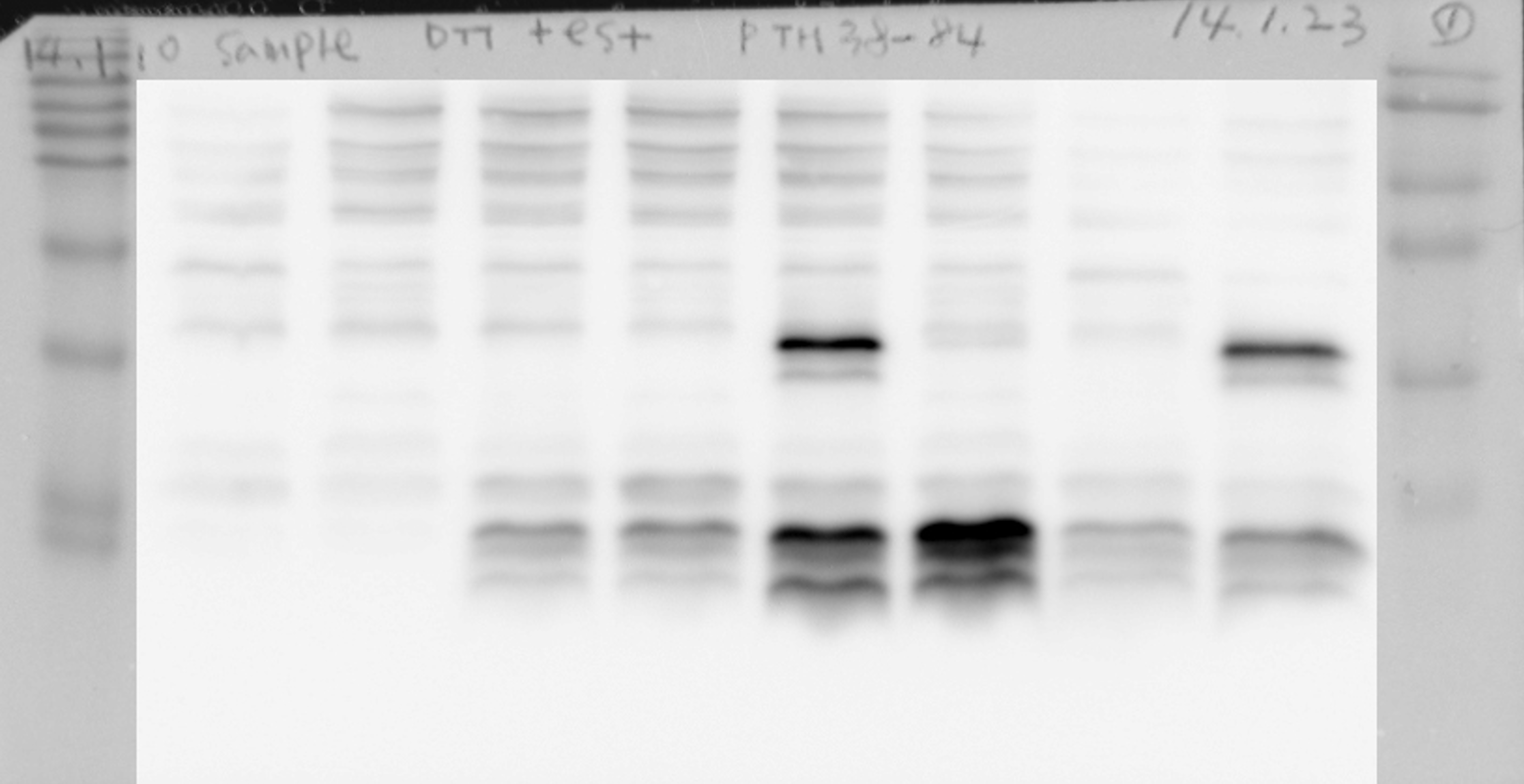

Supplement: Figure 1—figure supplement 2—source data 1. [file elife-97579-fig1-figsupp2-data1.zip › anti-PTH(39-84).tif]

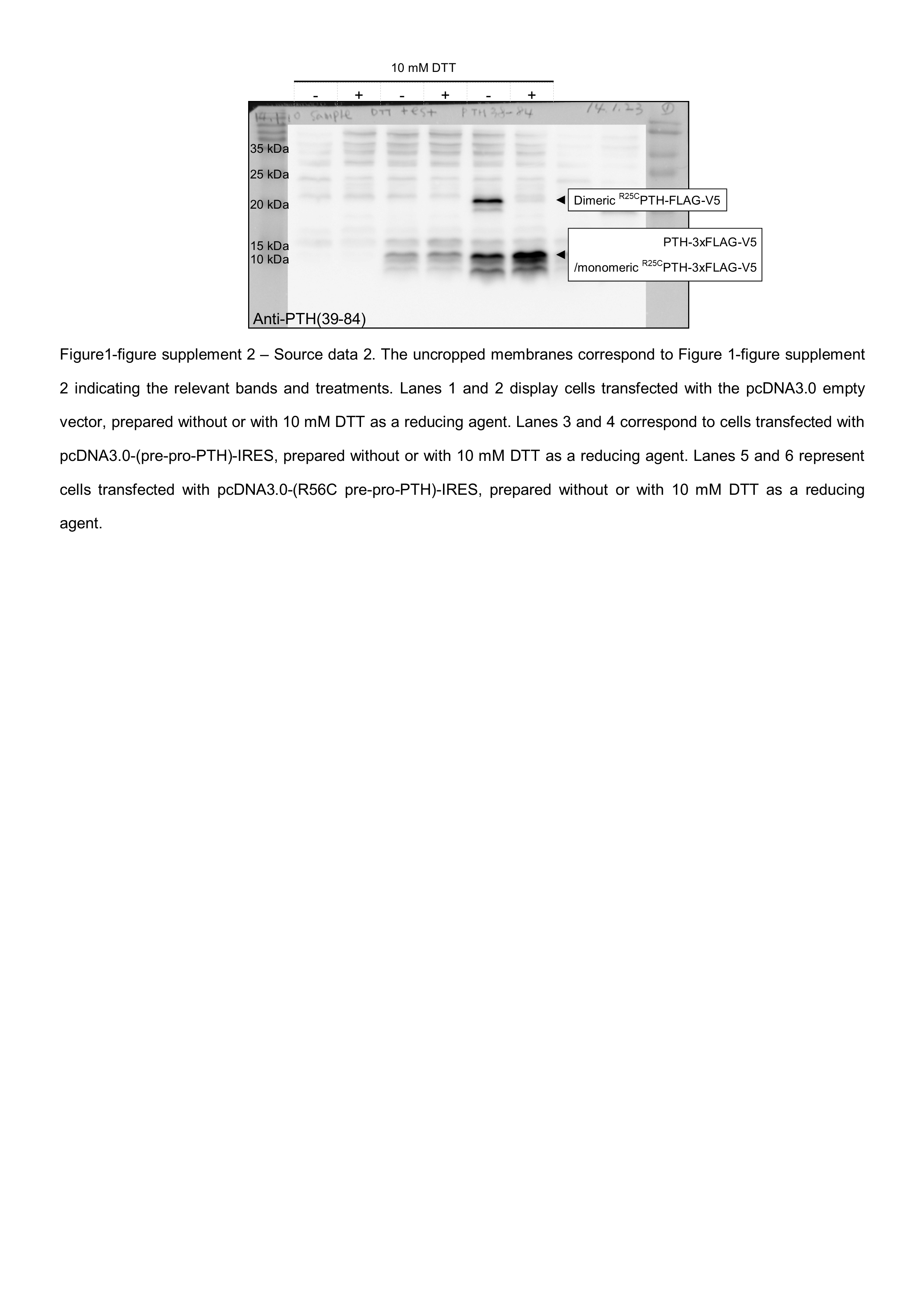

Supplement: Figure 1—figure supplement 2—source data 2. [file elife-97579-fig1-figsupp2-data2.zip › Figure supplement 2_Source data 2.tif]

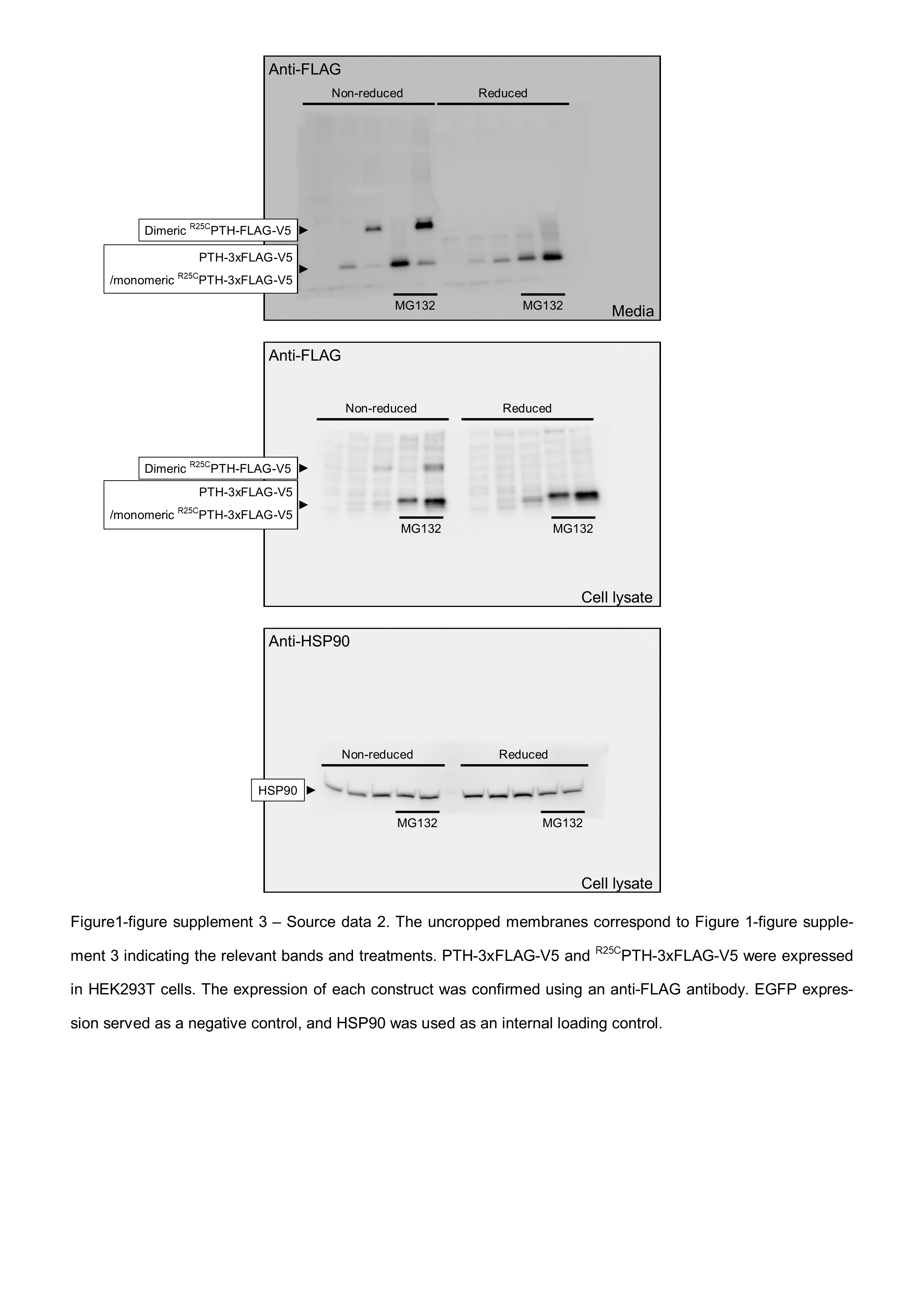

Supplement: Figure 1—figure supplement 3—source data 2. [file elife-97579-fig1-figsupp3-data2.zip › Figure supplement 3_Source data 2.tif]
