# Supplementary material for: Dimeric R25CPTH(1–34) activates the parathyroid hormone-1 receptor in vitro and stimulates bone formation in osteoporotic female mice
Source: eLife. 2025 Mar 28;13:RP97579. doi: 10.7554/eLife.97579 (PMC11952747; doi:10.7554/eLife.97579)
Supplement: Supplementary file 2. [file elife-97579-supp2.docx]

Supplementary File 2. List of primers used in this study

| Name | Sequence | Description |
| --- | --- | --- |
| PTH-Forward | 5’-GGGGACAACTTTGTACAAAAAAGTTGGCATGATACCTGCAAA  AGACATGGCTAAAG-3’ | Plasmid construction |
| PTH-Reverse | 5’-GGGGACAACTTTGTACAAGAAAGTTGGGTACTGGGATTTAGC  TTTAGTTAATACATTCACATCAG-3’ | Plasmid construction |
